# Phosphatidylinositol (4,5)-bisphosphate dynamically regulates the K_2P_ background K^+^ channel TASK-2

**DOI:** 10.1038/srep45407

**Published:** 2017-03-30

**Authors:** María Isabel Niemeyer, L. Pablo Cid, Marc Paulais, Jacques Teulon, Francisco V. Sepúlveda

**Affiliations:** 1 Centro de Estudios Científicos (CECs), Avenida Arturo Prat 514, Valdivia, Chile; 2 UPMC Université Paris 06, UMR_S 1138, Team 3 and INSERM, UMR_S 872, Paris, France

## Abstract

Two-pore domain K_2P_ K^+^ channels responsible for the background K^+^ conductance and the resting membrane potential, are also finely regulated by a variety of chemical, physical and physiological stimuli. Hormones and transmitters acting through Gq protein-coupled receptors (GqPCRs) modulate the activity of various K_2P_ channels but the signalling involved has remained elusive, in particular whether dynamic regulation by membrane PI(4,5)P_2_, common among other classes of K^+^ channels, affects K_2P_ channels is controversial. Here we show that K_2P_ K^+^ channel TASK-2 requires PI(4,5)P_2_ for activity, a dependence that accounts for its run down in the absence of intracellular ATP and its full recovery by addition of exogenous PI(4,5)P_2_, its inhibition by low concentrations of polycation PI scavengers, and inhibition by PI(4,5)P_2_ depletion from the membrane. Comprehensive mutagenesis suggests that PI(4,5)P_2_ interaction with TASK-2 takes place at C-terminus where three basic aminoacids are identified as being part of a putative binding site.

The phosphoinositide phospholipids (PIPs) are made of a water-soluble inositol head group and a phosphoglycerol moiety linking it to two fatty acid chains. PI(4,5)P_2_ phosphatidylinositol 4,5-bisphosphate is a minor component (<1%) of the inner leaflet of eukaryotic cell plasma membranes and yet is the most abundant of the PIPs populating it. Other PIPs, of which important ones are PI(3,4)P_2_ and PI(3,4,5)P_3_, are almost quantitatively negligible by comparison with PI(4,5)P_2_. Specific kinases and phosphatases regulate the phosphorylation state at various positions at the inositol ring and the level of PIPs in the membrane is maintained in a dynamic equilibrium. Negatively charged PIPs play prominent roles in the regulation of transmembrane protein function in eukaryotic cells, be it as purveyors of secondary signalling molecules generated through the action of receptor-activated enzymes, or through direct interactions with their target proteins. Rather prominent in the menu of membrane proteins directly controlled by PIPs are ion channels and transporters with which these phospholipids have convincingly been shown to interact physically, justifying the dubbing of this field in a recent review as “A frontier led by studies in ion channels”^1^.

PI(4,5)P_2_ is attacked by the enzyme phospholipase C (PLC) in response to activation of G-protein coupled receptors that signal through G_q/11_ Gα proteins. This produces soluble inositol 1,4,5-trisphosphate (IP_3_) and membrane-dwelling diacylglycerol (DAG), which respectively elicit a Ca^2+^ signal by release from intracellular stores and activation of protein kinase C (PKC). PI(4,5)P_2_, however, is a signalling molecule in its own right and its presence in the inner leaflet of the plasma membrane, initially recognised as essential for the activity of the Na^+^-Ca^2+^ exchanger and K_ATP_ channels^2^, is now known to be needed as a co-factor for many ion channels and transporters^3^,^4^. Virtually all K^+^ channels of the inwardly rectifying Kir family and several voltage-gated K_V_, and Ca^2+^-activated K_Ca_ channels require PIPs, generally PI(4,5)P_2_, for their activity^1^,^4^,^5^. Activation of G_q/11_ G-protein coupled receptors (GqPCRs) therefore generally lead to an inhibition of the channels associated to the concomitant decrease in membrane PI(4,5)P_2_. Whether the same occurs with K^+^ channels belonging to the two-P domain in tandem K_2P_ clade, has been controversial.

K_2P_ K^+^ channels are expressed widely and provide a leak pathway present in virtually all eukaryotic cells. K_2P_ channels are subjected to fine tuning by a variety of stimuli, including changes in membrane tension, voltage, temperature, extra- and intracellular pH, and G-protein coupled receptor activation, and play roles in a many important physiological processes^6^,^7^,^8^,^9^. The K_2P_ channel family has 15 mammalian members that fall into six groups: TWIK (weak inward rectifiers), TREK (lipid and mechanosensitive channels), TASK (acidification-inhibited), TALK (alkalinisation-activated), THIK (inhibited by halothane), and TRESK (spinal cord channels). Channels belonging to the TASK, TREK and TRESK subfamilies have been shown to be modulated by GqPCRs^10^. TASK channels TASK-1 and TASK-3^11^,^12^,^13^ and TREK-1 and TREK-2^11^,^14^,^15^,^16^ are inhibited by various hormones and transmitters signalling through GqPCRs. Exceptionally, TRESK channels are activated by GqPCR stimulation, a response mediated by increased intracellular Ca^2+^ that triggers a mechanism involving calcineurin^17^,^18^. TASK-1, TASK-3 and TREK-1 channels had been reported to be activated by PI(4,5)P_2_ and it was proposed that its hydrolysis underlies the inhibition by GqPCR agonists^19^. Other studies, however, have shown TASK and TREK channels to be insensitive to decreases in membrane PI(4,5)P_2_^20^,^21^,^22^. This suggests that although inhibited by GqPCR activation-elicited conversion of PI(4,5)P_2_ into DAG and IP_3_, the decrease in PI(4,5)P2 *per se* does not inhibit these K_2P_ channels. Instead it appears that DAG might play a central role in linking receptor activation and TASK and TREK channel modulation^11^,^23^.

The K_2P_ K^+^ channel known as TASK-2 (also K_2P_5.1)^24^,^25^ is involved in bicarbonate reabsorption in the proximal tubules of the kidney^26^, the central detection of CO_2_ and O_2_ by chemo-sensitive neurons implicated in breathing control^27^,^28^,^29^, and in the hearing function through an essential role in outer sulcus cells possibly in K^+^ recycling^30^. From a pathophysiological point of view, TASK-2 has been implicated in a predisposition to Balkan endemic nephropathy^31^ through a dominant negative missense mutation supressing function^32^,^33^, in the pathogenesis of multiple sclerosis and rheumatoid arthritis in humans^34^, and in murine inflammatory bowel disease^35^. TASK-2 is gated by extra- and intracellular pH, changes in cell volume and by Gβγ subunits of G proteins^36^,^37^,^38^,^39^. We have now explored the dependence on membrane phosphoinositides of the activity of TASK-2. Our results show that TASK-2 is strongly phosphoinositide-dependent and that this dependence probably involves an interaction of C-terminal positively charged residues with PI(4,5)P_2_. TASK-2 is a major player in a variety of physiological processes^24^,^25^ in which the phosphoinositide-dependence we have now uncovered might play a role. Our finding also points to the idea that K_2P_ channels, though departing in structure and regulation from other members of the K^+^ channel superfamily, do nevertheless share some of their regulatory characteristics.

## Results

### The activity of TASK-2 K^+^ channels is dependent on intracellular ATP and is decreased by positively charged polyvalent compounds

**Figure 1:**
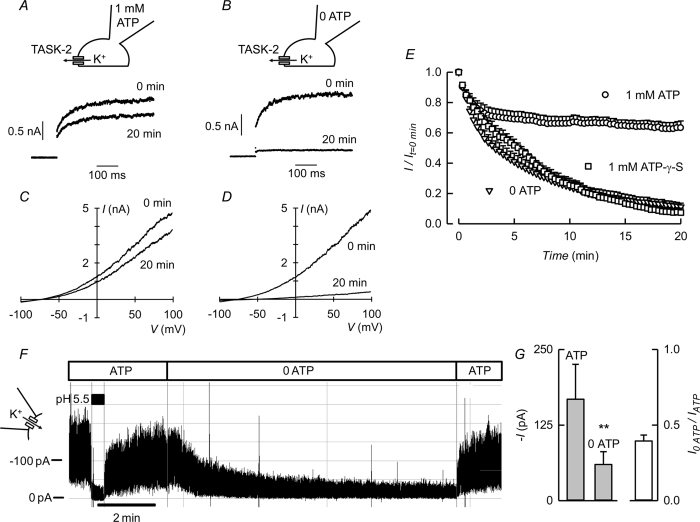
Effect of ATP removal on TASK-2-mediated currents. K^+^ currents recorded at 0 mV in HEK-293 cells expressing TASK-2 immediately after breaking into whole-cell recording (0 min) or after 20 min in cells dialysed with 1 mM ATP (**A**) or with the nucleotide omitted from the intracellular solution (**B**). The holding potential was −70 mV and the pulse was to 0 mV. (**C** and **D**) Corresponding current-voltage relations obtained from voltage ramps. (**E**) Time course of the K^+^ currents (means ± SEM) recorded at 0 mV in HEK-293 cells expressing TASK-2 after gaining access into the cells with pipette solutions containing 1 mM ATP (n = 10), no ATP (n = 9) or ATP-γ-S (n = 4). (**F**) Recording from an excised inside-out patch of membrane taken from a HEK-293 cell over-expressing TASK-2. The patch was held at −60 mV and at the indicated times intracellular 1 mM ATP was removed from the intracellular solution. During the time indicated by the black bar intracellular pH was decreased from 7.4 to 5.5. (**G**) Average currents measured before and after ATP removal (means ± SEM, n = 21). The observed decrease in current was statistically significant when measurements were compared with a paired test (**P < 0.001).

Certain classes of ion channel require the presence of intracellular ATP and deactivate when the intracellular aspect of isolated membranes where they reside is exposed to solutions lacking ATP, a phenomenon known as “run-down”. ATP is required for continued activity of TASK-2 that otherwise declines markedly in its absence or upon replacement by a non-hydrolysable analogue (Fig. 1). Whole-cell patch-clamp recordings of HEK-293 cells expressing TASK-2 yield typical K^+^ currents that persist in the presence of intracellular ATP (Fig. 1A and C) but markedly decline in its absence (Fig. 1B and D). The time course of current decrease shown in Fig. 1E has a half time of around 3 min and requires a hydrolysable nucleotide, as the non-hydrolysable analogue adenosine-5′-(γ-thio)-triphosphate (ATP-γ-S) was unable to support the TASK-2-mediated current. A similar result was obtained for TASK-2 channels in excised inside-out patches of membrane (Fig. 1F and G) that run-down reversibly upon removal of ATP from the solution bathing the intracellular aspect of the membrane. These patches, excised from cells overexpressing TASK-2 and recorded with low resistance pipettes, yielded robust inward K^+^ currents that were absent from untransfected cells and which obeyed to changes in intracellular pH exactly as expected from the typical TASK-2 behaviour (see Supplemental Fig. S1).

**Figure 2:**
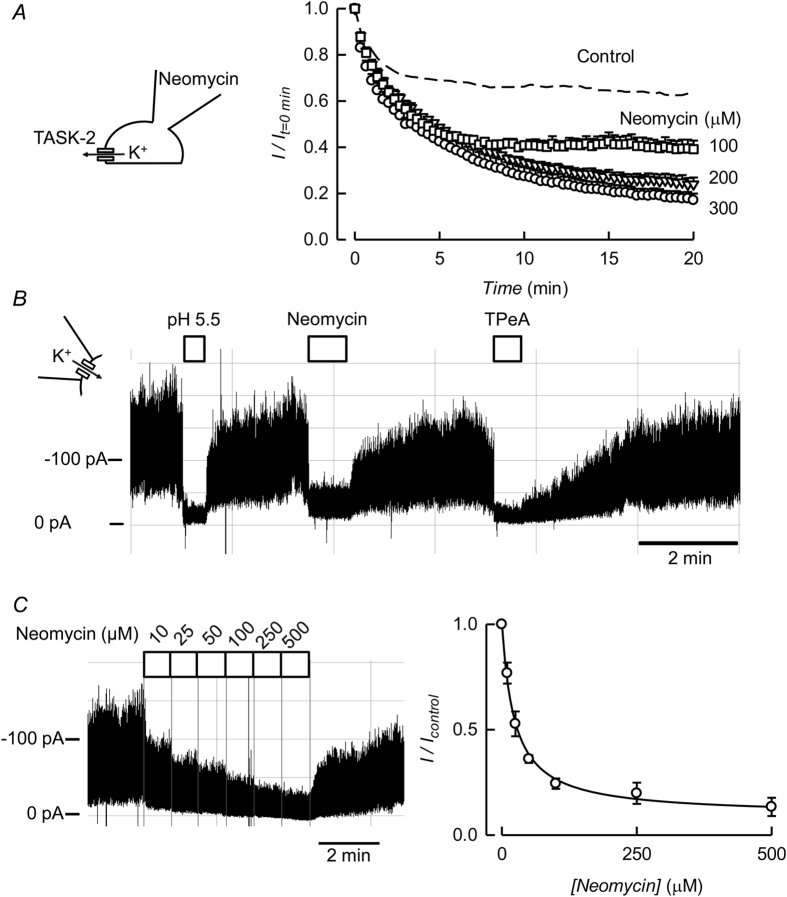
Effect of PI(4,5)P_2_ scavengers on TASK-2-mediated currents. (**A**) Time course of K^+^ currents (means ± SEM) recorded at 0 mV in HEK-293 cells expressing TASK-2 after gaining access into the cells with pipette solutions containing 100 (n = 3), 200 (n = 4) or 300 (n = 9) μM Neomycin. The recordings were made in the presence of 1 mM intracellular ATP. The control curve shown as a dashed line is from Fig. 1E. (**B**) Recording from an excised inside-out patch of membrane taken from a HEK-293 cell over-expressing TASK-2. The patch was held at −60 mV in the presence of 1 mM intracellular ATP. During the times indicated by the bars intracellular pH was decreased from 7.4 to 5.5, or 300 μM Neomycin or 100 μM TPeA were added. (**C**) Recording from an excised inside-out patch obtained under the same conditions as in (**B**), except that the concentration of neomycin in the solution bathing the intracellular aspect of the membrane was increased stepwise from 5 to 500 μM as shown. The graph shows the means of four experiments that gave an average IC_50_ for neomycin of 23.4 ± 2.7 μM (mean ± SEM, n = 4).

Run-down of ion channel activity in the absence of ATP is often an indicator of channel-dependence on membrane PIPs, most commonly PI(4,5)P_2_. PI(4,5)P_2_ is continuously hydrolysed and simultaneously replenished by enzymes that require ATP (and Mg^2+^)^1^,^2^. An important part of the ion channel-PIP interaction is electrostatic, involving the negatively charged PIP head and positive charges in the channel protein. Inhibition of channel activity by polyvalent cations is taken as evidence of masking PI(4,5)P_2_ negative charges, therefore disabling the interaction^40^. The polycationic antibiotic neomycin applied intracellularly inhibits TASK-2 activity (Fig. 2A) with a near maximum effect already at 200 μM with 100 μM yielding an intermediate effect. Neomycin also strongly inhibited TASK-2 activity in excised inside-out patches (Fig. 2B). The effect of neomycin at 300 μM was only slightly less marked than that seen during channel closure by acidification^38^ or by 50 μM of the intracellular pore blocker tetrapentyl ammonium (TPeA)^41^, which inhibits TASK-2 with an IC_50_ of ~2 μM (not shown). Figure 2C shows the effect of graded addition of neomycin applied intracellularly to an inside-out patch of membrane on TASK-2 current. The inhibitory effect was dose-dependent with an IC_50_ of 23.4 ± 2.7 (mean ± SEM) in four separate experiments.

### TASK-2 inhibition by activation of a Gq-coupled receptor

**Figure 3:**
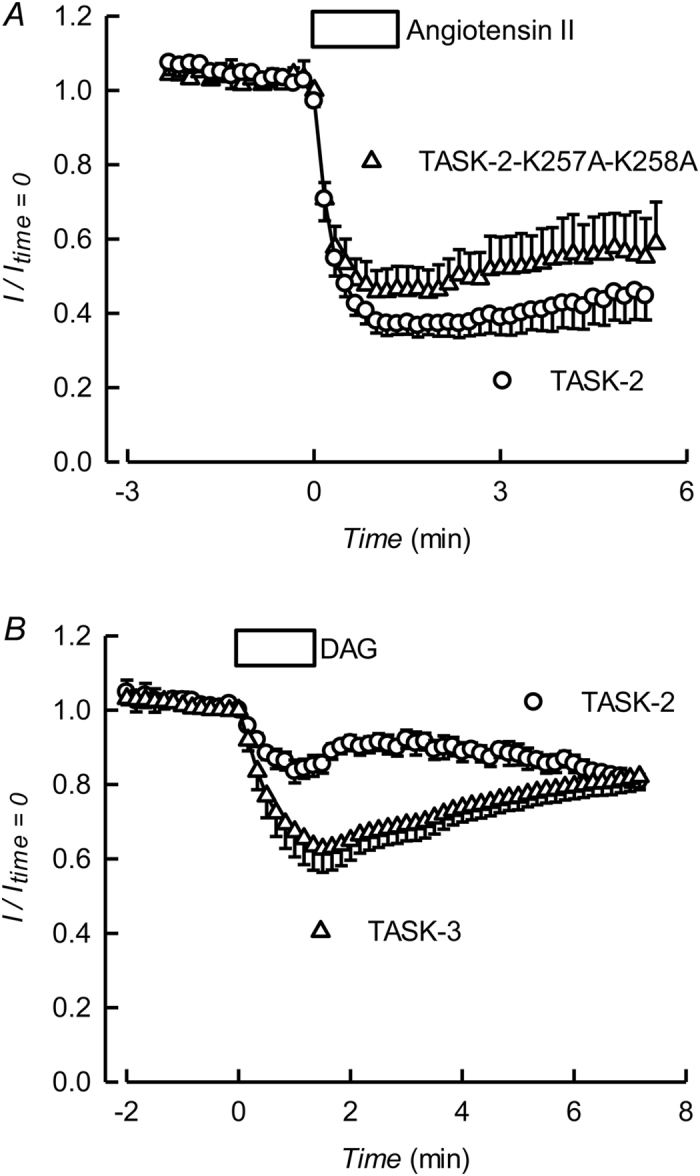
Gq protein coupled receptor inhibition of TASK-2 and minor effect of diacylglycerol. (**A**) Effect of activation of the angiotensin II receptor AT_1_ on TASK-2-mediated currents. HEK-293 cells were co-transfected with AT_1_ and TASK-2, or the TASK-2-K257A-K258A mutant. Current was monitored by brief pulses to 0 mV from a holding potential of −75 mV. Angiotensin II at a concentration of 1 μM was added during the indicated time. Results are means ± SEM of 6 experiments for TASK-2 and 4 experiments for TASK-2-K257A-K258A. (**B**) Effect of a diacylglycerol analogue assayed on TASK-2- and TASK-3-expressing cells. Current was monitored by brief pulses to 0 mV from a holding potential of −75 mV. The box labelled DAG indicates the addition of diacylglycerol short-chain analogue 1,2-dioctanoyl-*sn*-glycerol at 100 nM. Results are means ± SEM of 5 experiments for TASK-2 and 8 experiments for TASK-3.

It is well established that activation of GqPCRs is accompanied by PI(4,5)P_2_ metabolism leading to depletion in the membrane. This can cause inactivation of ion transporters or channels such as the M-type K_V_7.2/7.3 K^+^ channels that control the firing of neurones responsible for catecholamine secretion^42^,^43^,^44^,^45^. Consistent with a possible sensitivity to PI(4,5)P_2_, TASK-2 channels expressed with angiotensin II AT_1_ receptors are markedly inhibited by stimulation with angiotensin II (Fig. 3A). TASK-2 is inhibited by Gβγ subunits of G proteins and this effect is crucially dependent upon C-terminus lysine residues 257 and 258^39^. The angiotensin II effect was not significantly different in the Gβγ-insensitive TASK-2-K257A-K258A mutant (Fig. 3A).

The regulation of TASK-1 and -3 K_2P_ channels by GqPCRs was originally ascribed to a direct dependence on PI(4,5)P_2_^19^ but this view has now been challenged^20^,^21^. The most recently published evidence supports a hypothesis according to which GqPCR inhibition of TASK-1 and TASK-3 is exclusively due to released diacylglycerol (DAG) directly acting on the channel protein, without any role for the concomitant reduction in PI(4,5)P_2_^23^. An inhibitory effect of DAG has also been shown for TREK-1 and found important in its regulation by GqPCRs^11^. DAG, however, only caused a minor effect on TASK-2, in contrast with the strong inhibition it produces on TASK-3 (see Fig. 3B), suggesting that it is not the main drive behind AT_1_-dependent inhibition of TASK-2.

### Plasma membrane PI(4,5)P_2_ depletion by a voltage-sensitive phosphatase inhibits TASK-2 activity

**Figure 4:**
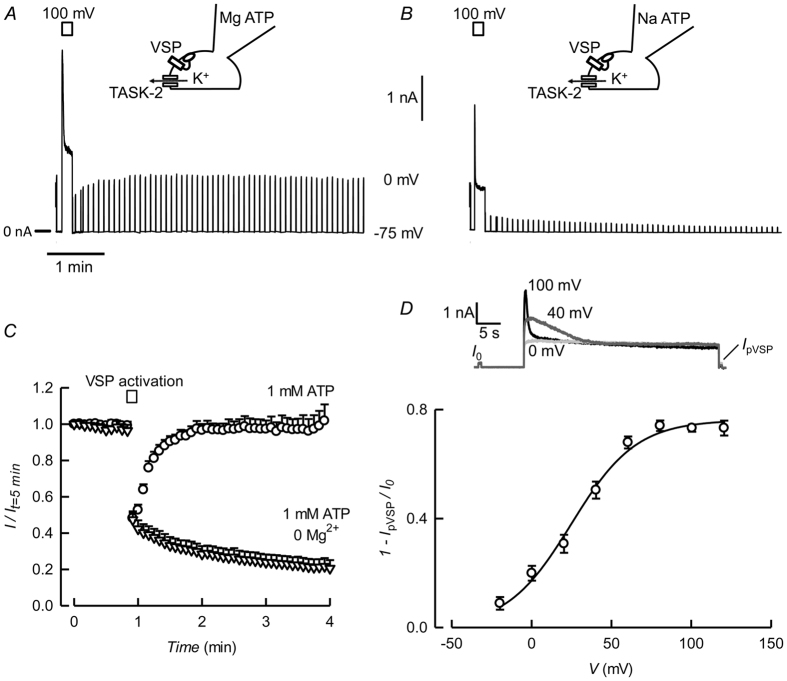
Sensitivity of TASK-2 to co-expressed DrVSP activation. (**A**) Representative trace of a current recording from a HEK-293 cell co-expressing TASK-2 and DrVSP. The potential was held at −75 mV and 300 ms pulses to 0 mV were delivered at 5 s intervals. DrVSP was activated with a 10 s depolarisation to 100 mV. The intracellular solution contained 1 mM ATP and 1 mM Mg^2+^. (**B**) As in (**A**), but with Mg^2+^ omitted from the intracellular solution. (**C**) Average currents for experiments as in A and B. Results are the means ± SEM of 7 (circles) and 6 (triangles) experiments. (**D**) Voltage-dependence of DrVSP activation on TASK-2-meadiated current. The results are means ± SEM of 7 experiments and the continuous line is a fit of a Boltzmann equation to the data.

For a more direct test of the hypothesis that TASK-2 is PI(4,5)P_2_-sensitive we have used DrVSP, a voltage sensitive phosphatase from *Danio rerio*, that removes 5-phosphate groups from PI(4,5)P_2_ thereby depleting the membrane of this PIP rapidly and efficiently^46^. When TASK-2 is expressed together with DrVSP in HEK-293, strong depolarisation leads to a marked inhibition of the current that is not present in cells transfected with TASK-2 alone (see Supplementary Fig. S2). The decline in channel activity elicited by DrVSP activation by depolarisation is reversible upon return to a negative potential (Fig. 4A), but only in the presence of Mg^2+^ (Fig. 4B and C). This is to be expected if endogenous, Mg^2+^-dependent PI-5-kinase were resynthesising PI(4,5)P_2_ from PI(4)P once the activity of DrVSP is discontinued.

The magnitude and speed of current inhibition by DrVSP activation are dependent on voltage (Fig. 4D). The degree of current inhibition rises with depolarisation with a V_1/2_ value of 20 ± 2.2 mV. This voltage-sensitivity has been shown to be the consequence of the phosphatase titrating PI(4,5)P_2_ in the plasma membrane as the PIP will decrease with increasing depolarisation^44^,^47^. On the basis of its voltage sensitivity in these DrVSP co-expression experiments, the apparent affinity of TASK-2 for PI(4,5)P_2_ should be close to that of Kv7.3 and Kir2.1^47^.

### Activation of TASK-2 channels by intracellular application of phosphoinositides

**Figure 5:**
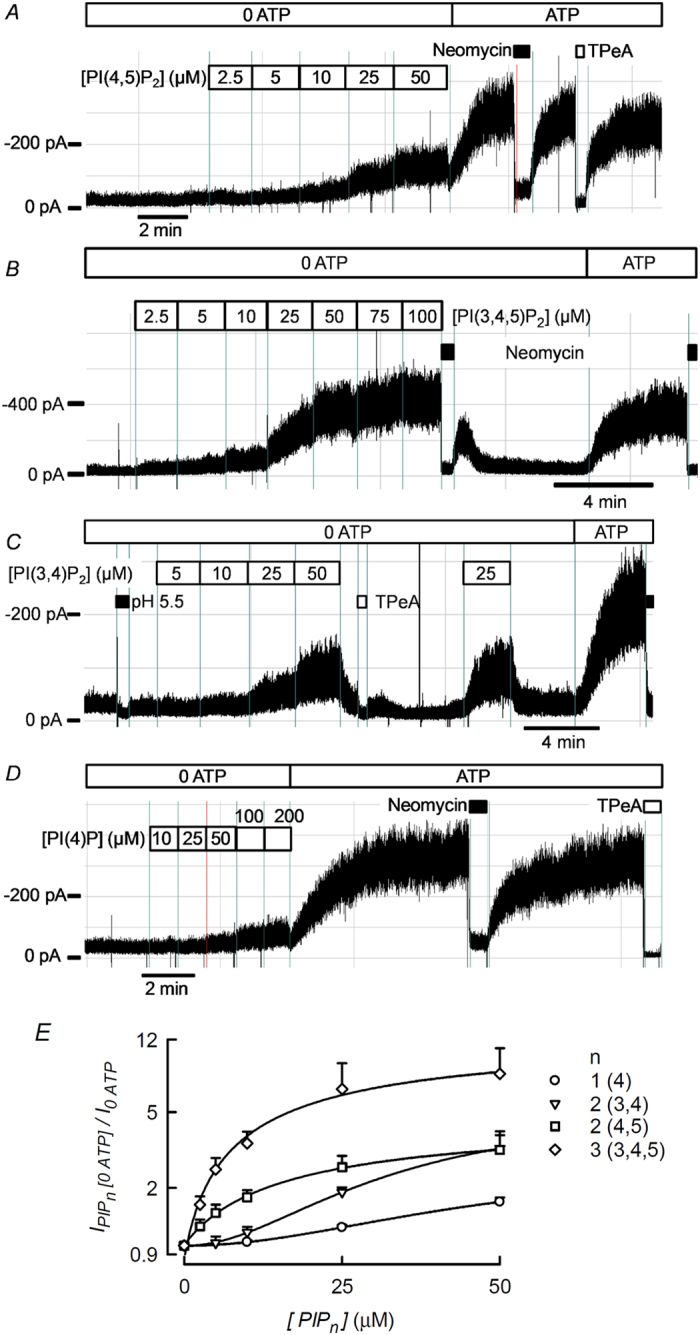
Concentration dependence of the effect of some phosphoinosides on TASK-2 activity. (**A**) Recording from an excised inside-out patch of membrane taken from a HEK-293 cell over-expressing TASK-2. The patch was held at −60 mV and was initially in absence of ATP for at least 5 min, a condition that inhibits the current. The effect of PI(4,5)P_2_ in its diC8 is shown. The PIP was added at increasing concentrations from 2.5 to 50 μM. ATP at 1 mM was then readmitted and the effects of 300 μM Neomycin and 100 μM TPeA tested. (**B**,**C** and **D**) Similar experiments but using diC8 PI(3,4,5)P_3_, PI(3,4)P_2_ and PI(4)P respectively. Other details as in (**A**). (**E**) Summary of the effects of the four PIPs tested. The effects are presented as the average current recorded at a given concentration of the PIP normalised to that in its absence, with all measurements taken in the absence of ATP. Lines are fitted rectangular hyperbolae for PI(3,4,5)P_3_ and PI(4,5)P_2_, and Hill equations with n_H_ of 2 for PI(3,4)P_2_ and PI(4)P. Maximal fold effects from these fits were (means ± SEM) 11.6 ± 1.6, 3.7 ± 0.2, 4.3 ± 0.4 and 1.4 ± 0.4 respectively for PI(3,4,5)P_3_, PI(4,5)P_2_, PI(3,4)P_2_ and PI(4)P. The corresponding K_0.5_ values were 29.8 ± 10.2, 34.9 ± 3.3, 48.4 ± 4.6 and 50.6 ± 1.3 μM. Points in the graph are means ± SEM of 3–8 experiments.

If the rundown of TASK-2 in excised membrane patches in the absence of ATP is due to a decline in PI(4,5)P_2_, a recovery of the current would be expected to occur by addition of exogenous phosphoinositide. The experiments in Fig. 5A–D test the ability of PI(4,5)P_2_ and other phosphoinositides, all in their soluble DiC8 form, to stimulate TASK-2 after its activity has decreased in the absence of ATP. All phosphoinositides tested had some stimulating effect on TASK-2-mediated currents when added to inside-out excised patches, but they varied markedly in their potency. Largest effect was obtained with PI(3,4,5)P_3_ whilst the response to PI(4)P was quite small. Robust, but intermediate effects were exerted by PI(4,5)P_2_ and PI(3,4)P_2_. The effects are compared quantitatively in Fig. 5E where the EC_50_ values can be seen to rank in the order PI(3,4,5)P_3_ < PI(4,5)P_2_ < PI(3,4)P_2_ < PI(4)P (see Fig. 5E legend). Interestingly, the dose response curves for PI(3,4)P_2_ and PI(4)P were best fitted by Hill equations with n_H_ of 2, whilst those for PI(3,4,5)P_3_ and PI(4,5)P_2_ were rectangular hyperbolae. The sensitivity of TASK-2 to the diC8 form of PI(4,5)P_2_ would indicate an apparent affinity similar to that of Kir2.3^48^ or the heteromer formed by Kv7.2 and Kv7.3^49^.

Addition of ATP after treatment with PIPs appeared to elicit higher currents than those under maximal PIP stimulation. This impression is quantified in Supplementary Fig. S3. The effect of ATP readmitted to the intracellular aspect of inside-out patches was much larger (5.6-fold) than the maximal effect elicited by PI(4)P. The effect of ATP was also larger than the maximal activation with PI(3,4)P_2_ or PI(4,5)P_2_ but this only 1.4- and 1.7-fold respectively, whilst that of PI(3,4,5)P_3_ was similar to that elicited by ATP. Assuming that ATP restores normal levels of endogenous membrane PI(4,5)P_2_ and that this is the natural TASK-2 activator phosphoinositide, we speculate that PI(4)P with only one phosphate in its head group is not capable to emulate its efficacy. It is possible that the short-chain diC8 forms of PI(3,4)P_2_ or PI(4,5)P_2_ used here do not faithfully supplant the potency of the native, long-chain PI(4,5)P_2_. This might be compensated in the case of PI(3,4,5)P_3_ on a purely electrostatic basis. Other, unidentified ATP-dependent factors contributing to TASK-2 activity may also intervene to explain our results, but these, if present, will need further work to unravel.

### Identifying TASK-2 residues responsible for interaction with membrane PI(4,5)P_2_

**Figure 6:**
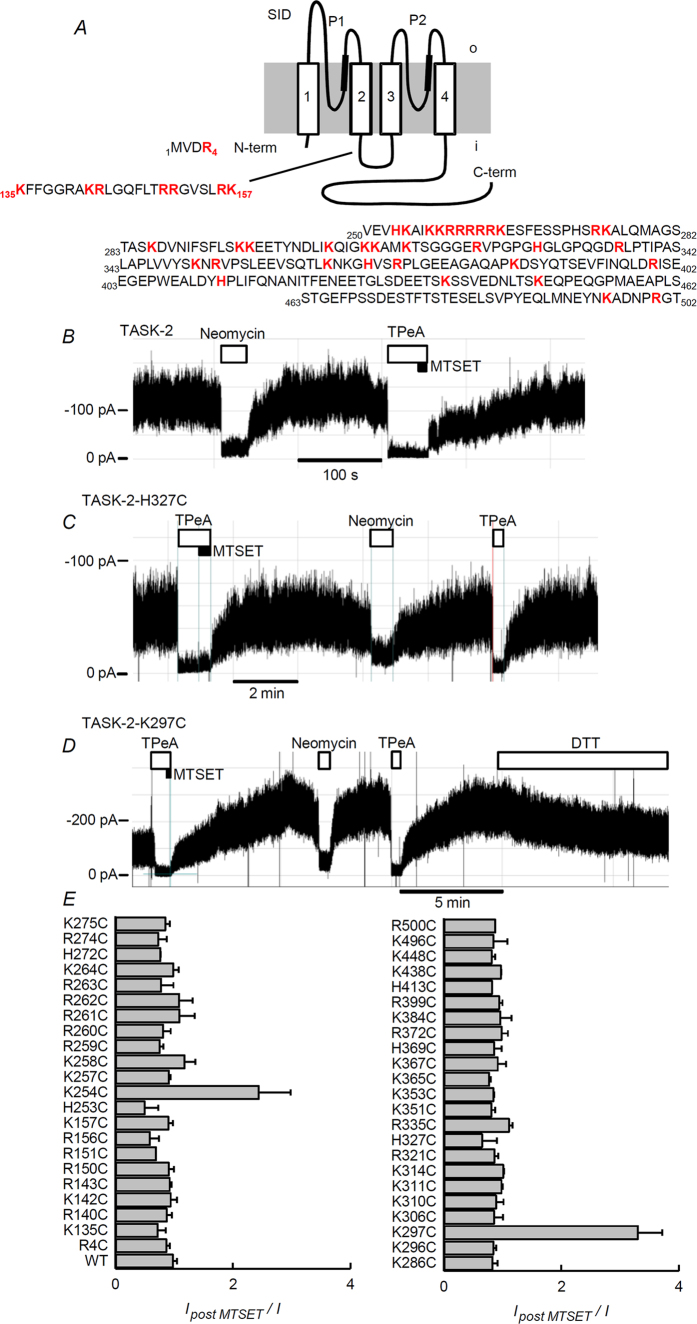
Scanning of potential sites of interaction of TASK-2 with PI(4,5)P_2_ by cysteine mutagenesis of intracellular basic residues. (**A**) Schematic representation of TASK-2 topology showing the sequences of segments of the protein predicted to be intracellular or intracellular-facing. Basic residues are highlighted in red. (**B**) Recording from an excised inside-out patch of membrane taken from a HEK-293 cell over-expressing TASK-2. The patch was held at −60 mV in the presence of 1 mM ATP. At the intervals indicated by the boxes the intracellular aspect of the membrane was exposed to 300 μM Neomycin and then to 100 μM TPeA. At the end of the TPeA period the patch was additionally exposed to 1 mM MTSET as shown by the black box. (**C** and **D**) experiments as in (**B**), but with inside-out membrane patches obtained from cells expressing the mutants TASK-2-H327C and TASK-2-K297C respectively. In D the reducing agent DTT was also added at 2 mM. (**E**) Summary of experiments comparing the current before and after treatment with MTSET. The only mutants differing significantly from WT TASK-2 were TASK-2-K254 C and TASK-2-K297C (P < 0.001 by Bonferroni-corrected t-test).

The interaction between PI(4,5)P_2_ and the proteins it regulates have been shown to be electrostatic in nature^50^, and naturally-occurring mutations affecting basic residues in Kir channels decrease their affinity for PI(4,5)P_2_ and lead to channelopathies^51^. If the same principle applied to TASK-2, replacement of positively charged residues facing intracellularly (identified in Fig. 6A) by the neutral cysteine residue should reduce its activity. The reactivity of cysteine affords the possibility of using charge, and activity restoration with positively-charged small methanethiosulfonate reagents to identify basic residues important for PI(4,5)P_2_ interaction.

**Figure 7:**
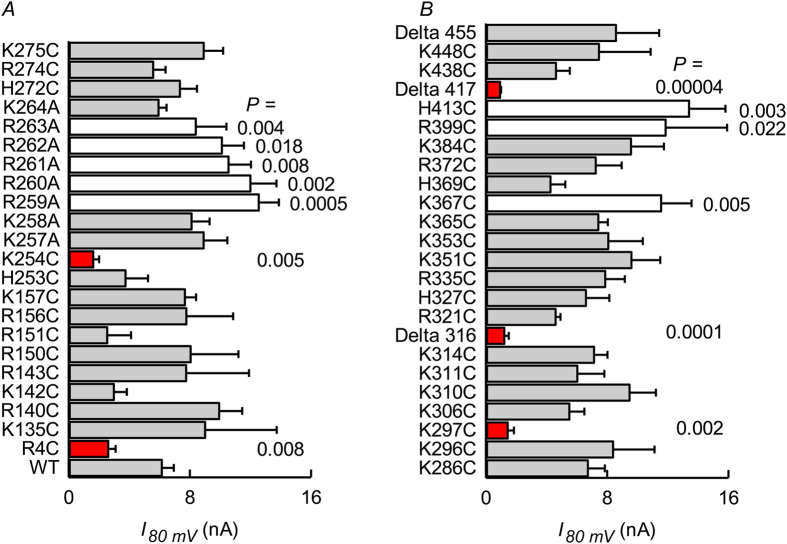
Effect of neutralising positively charged residues on the current mediated by TASK-2. Results of assays by whole-cell patch-clamp recordings of TASK-2 channels carrying the indicated point mutations, and also some deletion mutants, are shown. Those yielding significantly smaller currents than the WT TASK-2 are identified in red. Mutants giving what appeared as significantly larger currents are identified by the empty columns. The P values quoted are for t-test comparisons giving a significant difference with WT TASK-2.

A survey of the whole-cell currents associated to expression of the different mutants gave highly variable results (Fig. 7). Only three point mutants, TASK-2-R4C, -K254C and -K297C, showed significantly decreased currents with respect to WT TASK-2. Several mutants exhibited significant increases in current. Of interest are those present in the stretch between positions 259 and 263, belonging to a remarkable basic amino acid-rich region in the early portion of the C-terminus. These might be gain-of-function mutants, but their identification as such will have to await further studies particularly of their expression levels. As variability could in general be due to alterations in expression levels or other unknown effects rather than to alterations in the strength of a putative channel-PI(4,5)P_2_ interaction, all cysteine mutants were tested for increases in activity upon methanesulfonate reagent treatment. Figure 6B–D show examples of recordings from inside-out patches from cells expressing non-mutated TASK-2 or the H327C and K297C mutants. Neither WT nor H327C channel activity is affected by brief intracellular exposure to [2-(trimethylammonium)ethyl]methanethiosulfonate (MTSET, used in the presence of TPeA, see Supplementary material). The activity of TASK-2-K297C on the other hand is markedly enhanced by MTSET treatment and this effect was reversed, at least partially by addition of the reducing agent dithiothreitol (DTT). A summary of our systematic exploration of all intracellularly facing basic residues of TASK-2 is shown in Fig. 6E. The survey suggests that K297 is a determinant of the interaction of the channel with membrane PI(4,5)P_2_. A second residue that appears of importance is K254 as TASK-2-K254C mutant is also significantly activated by the MTSET treatment (Fig. 6E). TASK-2-R4C that like TASK-2-K297C appeared to show low activity, was not affected by MTSET treatment.

**Figure 8:**
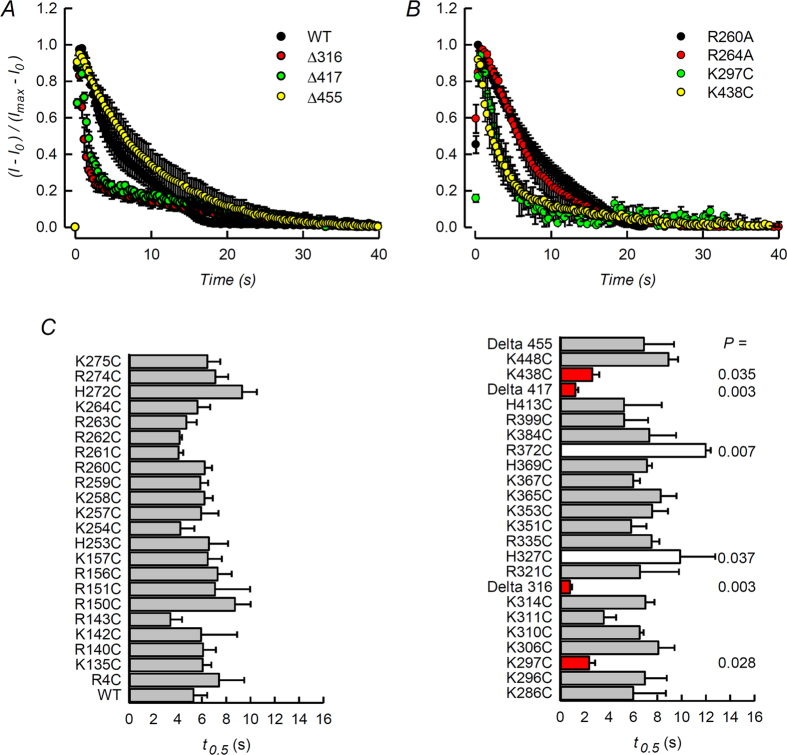
Inhibition of various mutants of TASK-2 by co-expressed DrVSP activation. (**A** and **B**) Averages of current traces recorded from a HEK-293 cell co-expressing WT TASK-2 or various truncated or point mutated channels together with DrVSP. The potential was held at −75 mV and a pulse to 80 mV with a duration of 40 s was given to activate DrVSP. The graphs report averages with SEMs of 5–10 experiments. The intracellular solution contained 1 mM ATP and 1 mM Mg^2+^. (**C**) Summary of results of effects of DrVSP activation on currents mediated by WT or mutant TASK-2 channels. The columns report the time taken for currents to decrease by 50% (t_0.5_) after stepping the voltage from −75 mV to 80 mV. The results are means ± SEM of 3–10 experiments. In red mutants that gave t_0.5_ values significantly smaller than WT with P < 0.05. White columns identify mutants that appeared to be inhibited more slowly than WT TASK-2.

It would appear from the experiments just described that from the positively charged residues candidate in TASK-2 that are expected to face the intracellular milieu only two, C-terminus-located lysines would be responsible for an interaction with membrane PIPs. A limitation in the approach, however, is that there might be amino acids important in the interaction but that are not accessed by the MTSET reagent during the course of the experiment. Alternatively the charged residue might be necessary in a function unrelated to a phosphoinositide interaction. A further test of the hypothesis is to assess the ability of DrVSP to inhibit WT and mutant TASK-2 channels to detect possible changes in the affinity of TASK-2 for PI(4,5)P_2_. It is well known that differences in the kinetics of deactivation of K^+^ channels by VSPs reflect differences in the affinities of said channels for PI(4,5)P_2_. For example deactivation of Kir2.1-R228Q, a mutant channel with independently demonstrated weakened interaction with PI(4,5)P_2_ as compared with WT Kir2.1, occurs with a faster time course upon VSP activation than in the WT channels^47^. We first examined TASK-2 channels carrying deletions in their C-terminus eliminating different portions of the C-terminus: Δ316, Δ417 and Δ455. Results are shown in Fig. 8A and reveal that deactivation by DrVSP was markedly faster than for WT TASK-2 in truncated mutants Δ316 and Δ417, but not in Δ455. This might imply that the stretch of tail between residues 316 and 455 contains amino acids involved in the interaction, while those beyond 455 are not relevant. Unfortunately this excludes only residues K496 and R500, and does not discard those located upstream of residue 316. We undertook the examination of the single point mutants and Fig. 8B shows two mutants exhibiting slow time course of DrVSP inhibition, R260A and R264A, a kinetics that did not differ from that seen when using WT TASK-2. A much faster current inhibition by DrVSP activation, however, was seen on the currents mediated by the K297C and R438C mutants of TASK-2 (Fig. 8B) suggesting that the absence of these residues weakens a putative interaction of the channel with membrane PIP. A systematic test of the remaining candidate mutants revealed that, besides the aforementioned truncated mutants, only these two point mutants showed significantly faster rates of DrVSP-induced channel deactivation (identified by red bars in Fig. 8C).

Finally, we attempted a comparison between the potency of inhibition by neomycin between mutants and the WT channel as a means to further test changes in the TASK-2-PI(4,5)P_2_ affinity. The currents expressed in isolated patches by TASK-2 mutants R254C and K297C were too small to yield reliable measurements of IC_50_ for neomycin inhibition. That mediated by TASK-2-R438C was sufficiently large, however, and an IC_50_ value of 8.9 ± 1.5 μM (see Supplementary Fig. S7) was obtained. This was significantly lower that measured using WT TASK-2 (23.4 ± 2.7 μM, see Fig. 2) as tested using t-test (P = 0.003).

## Discussion

The present results demonstrate that background K_2P_ K^+^ channel TASK-2 requires the presence of membrane phosphoinositides for activity and that this dependence probably involves interaction of C-terminal positively charged residues with PI(4,5)P_2_. Several observations that are normally taken as diagnostic of a role for PI(4,5)P_2_ as an important cofactor to channel function^3^,^4^ are fulfilled by the data presented here for TASK-2. These can be summarised as follows: (a) The activity of TASK-2 expressed heterologously rapidly runs down in the absence of intracellular ATP, or when this is replaced with a non-hydrolysable analogue, either in whole-cell recordings or in isolated membrane patches; (b) TASK-2 channels recover from this rundown by addition of exogenous PI(4,5)P_2_ in excised membrane patches; (c) Neomycin and poly-lysine, polycation PI scavengers, also inhibit TASK-2 activity in the presence of intracellular ATP; (d) TASK-2 channels are strongly inhibited by activation of coexpressed voltage-sensitive phosphatase DrVSP that removes 5-phosphate groups from PI(4,5)P_2_; (e) Recovery from DrVSP-catalysed PI(4,5)P_2_ hydrolysis is inhibited in the absence of Mg^2+^ suggesting that the levels of PI(4,5)P_2_ re maintained by a dynamic equilibrium of hydrolysis and resynthesis requiring Mg-ATP.

The requirement for PI(4,5)P_2_ of TASK-2 is of low selectivity as all phosphoinositides tested had some stimulating effect on TASK-2. The most potent effect was that of PI(3,4,5)P_3_ and the effect of PI(4)P was rather weak. Phosphoinositides in general are minor components of the phospholipid population of cells. For example PI(4)P and PI(4,5)P_2_ make only 0.2–1% of the total cellular phospholipids. Both PI(3,4,5)P_3_ and PI(4,5)P_2_ are found in the plasma membrane but the abundance of the first is only 2–5% of that of PI(4,5)P_2_^52^ suggesting that even being more potent in activating TASK-2, its contribution to TASK-2 activity is only minor. PI(4)P is abundant in the Golgi complex while PI(3,4)P_2_ is found in the plasma membrane and the early endosome compartment^53^. The relatively weak effect of PI(4)P probably explains why DrVSP activity has a potent effect on TASK-2 even when considering that DrVSP action converts PI(4,5)P_2_ into PI(4)P. This contrasts with the lack of effect of the same phosphatase on TREK-1^22^. This result, apparently surprising in view of the well characterised requirement of an interaction of a polybasic motif in its C terminus and the most abundant phosphoinositide in the plasma membrane, PI(4,5)P_2_^54^,^55^, has been explained on the basis of a lack of phospholipid specificity in the effect on TREK-1^22^. It is puzzling that PI(4,5)P_2_ apparent affinity of TASK-2 judged from the voltage-dependence of DrVSP effect^47^ would put this K_2P_ channel in a high affinity category together with Kir2.1^51^ and Kv7.3^49^,^56^ (diC8 PI(4,5)P_2_ EC_50_ 3–6 μM). Affinity for the long-chain PI(4,5)P_2_ that is dephosphorylated by DrVSP may, however, be different from that for shorter diC8 form.

Consistent with a main requirement for PI(4,5)P_2_ as a cofactor for TASK-2 activity is its inhibition by activation of coexpressed Gq-coupled angiotensin II AT_1_ receptors. K_2P_ channels TASK1, TASK3, TREK1 and TREK2 are inhibited by agonists that activate Gq proteins both in native and recombinant systems^11^,^12^,^57^. Initially, it was hypothesised that inhibition was due to the PI(4,5)P_2_ decline due to activation of Gq-coupled receptors^19^, but this view was contradicted by further work^20^,^21^. Phospholipase Cβ activation leads to a decrease in membrane PI(4,5)P_2_ and concomitant DAG production, which has been proposed as the mediator of the receptor-dependent inhibition of TREK-1 and -2^11^ and TASK-1 and -3^23^ channels. Use of DAG here reveals only a minor effect on TASK-2 suggesting that it is not the main signalling molecule linking receptor activation of channel inhibition. As TASK-2 is Ca^2+^-independent we surmise that the receptor effect is due to a decrease in PI(4,5)P_2_. The inhibition of TASK-2 associated to GqPR activation has not been reported before and its physiological relevance remains to be explored.

What are the molecular determinants responsible for the interaction of TASK-2 with membrane PI(4,5)P_2_? With the hypothesis that the interaction between TASK-2 and PI(4,5)P_2_ is electrostatic and requires the presence of intracellularly-facing positively charged residues, we proceeded to a systematic cysteine mutagenesis to determine if any of these amino acids took part in the binding. We reasoned that neutralisation of a putative interacting residue should lead to a decrease in the current the channel mediates, followed by an increase in current upon charge restoration after reaction with the MTSET reagent. Furthermore an acceleration of the rate at which DrVSP is capable of inhibiting the remaining current ought to occur because of a weakened interaction between the channel and PI(4,5)P_2_. The information for residues identified by these criteria is summarised in Table 1. It is seen that only K297 fulfils all three criteria and on this basis it is proposed that this residue is essential for the TASK-2-PI(4,5)P_2_ interaction. K254C exhibits both low current and activation by MTSET, but puzzlingly TASK-2-K254C shows unaltered kinetics of inhibition by DrVSP. Finally, mutation R438C accelerates the inhibition of TASK-2 by DrVSP but it is not affected by MTSET, which might reveal an accessibility problem. This mutant, however, is inhibited by lower concentrations of neomycin than WT TASK-2, suggesting that it is a genuine participant in the channel-PI(4,5)P_2_ interaction. R4C, on the other hand, only exhibits a diminished current and this could be due to the mutation altering the expression level of the channel or an unrelated functional property. In summary we conclude that three residues, K254, K297 and R438 all participate in the TASK-2-PI(4,5)P_2_ interaction, with the strongest evidence for K297. Interestingly, K297, together with K257 and K258, had been identified previously as possibly implicated in the inhibition of TASK-2 by Gβγ subunits of heterotrimeric G-proteins, but K297 was shown not to be required for Gβγ binding that is mediated by the K257/258 site only^39^.

The number of residues that appear involved in the PI(4,5)P_2_ sensitivity of TASK-2 contrasts with that found in channels such as Kir2.1 for which five arginines, four lysines and one histidine are important^51^. Recently discovered PI(4,5)P_2_ binding structures for Kir2.2 and Kir3.2 are made up of six positively charged amino acids that interact with the negatively charged head group of PI(4,5)P_2_ in each of four subunits of the tetramer^58^,^59^,^60^. These differences in interacting residues between TASK-2 and the Kir channels might produce marked differences in PI(4,5)P_2_ binding affinities. However, although we have an idea of the EC_50_ of the PI(4,5)P_2_ effect on TASK-2, we ignore the mechanism by which it affects gating and therefore we cannot infer an affinity. A better idea of the strength of the TASK-2-PI(4,5)P_2_ interaction can be gathered from the ability of neomycin, a PI(4,5)P_2_-screening compound, to inhibit the channel. Neomycin inhibits TASK-2 with an IC_50_ of around 20 μM, putting TASK-2 in a group with Kir4.1/Kir5.1 that has a neomycin IC_50_ of 110 μM^61^ and therefore classifiable as of low PI(4,5)P_2_ affinity in a Kir context^62^. Based on the EC_50_ for activation by diC8 PIP(4,5)P_2_, TASK-2 “affinity” would be intermediate and similar to that of the Kv7.2/Kv7.3 complex^49^. However, judged by the V_0.5_ of the VSP effect it would be close to that of the higher affinity Kv7.3^47^.

TASK-2 has also been shown to take part in the short term cell volume regulation process known as RVD, regulatory volume decrease^36^. A role for TASK-2 in RVD has been demonstrated in kidney tubule cells^63^ and in T and B immune-cells^64^,^65^. RVD occurs by acute parallel modulation of K^+^ and Cl^−^ conductances^66^ but the mechanism linking changes in cell volume to ion channel modulation have remained elusive. Neutralisation of K297 (together with K298) markedly decreases the sensitivity of TASK-2 to changes in cell volume^39^. This suggests that a modulation of the TASK-2-PI(4,5)P_2_ interaction might be important in commanding TASK-2 function in RVD. A possible mechanism for this action is that suggested for the voltage-dependent K^+^ channel made up by the complex KCNQ1/KCNE1^67^ that responds to changes in cell volume through dilution/concentration of intracellular polyamines and Mg^2+^ owing to their screening PI(4,5)P_2_ negative charges. A similar mechanism would explain the osmosensitivity necessary for TASK-2 to play a role in RVD. The EC_50_ for PI(4,5)P_2_ effect on KCNQ1/KCNE1 has been reported at 5 μM^68^, much lower than that for TASK-2 measured here at 35 μM. More importantly however, and discussed above, TASK-2 interaction with membrane PI(4,5)P_2_ is probably relatively weak as evidenced by competition with neomycin. On that basis we would postulate a high sensitivity of TASK-2 to native cellular polycationic compounds such as polyamines that could couple volume changes to channel activity though modulation of TASK-2-PI(4,5)P_2_ interaction.

In conclusion, TASK-2 is an example of a K_2P_ channel that depends on an interaction with membrane PI(4,5)P_2_ for activity and that is capable of being dynamically modulated by changes in the levels of this phospholipid in the membrane or by conditions that alter its interaction with the channel. Mutagenesis experiments suggest that interaction occurs with TASK-2 C-terminus where three basic aminoacids are identified as potentially important in a putative binding site. The apparent affinity of TASK-2 for membrane PI(4,5)P_2_ is low and it is conceivable that changing competition by intracellular polycationic compounds might mediate the previously reported sensitivity of TASK-2 to changes in cell volume. A decrease in PI(4,5)P_2_ by activation of Gq-PCRs is also probably responsible for TASK-2 inhibition. More generally, the dependence of TASK-2 upon PI(4,5)P_2_ might be important in the various physiological and pathophysiological scenarios in which it is proposed to be involved.

## Methods

### Plasmid construction and transfections

*Mus musculus* TASK-2 (GenBank accession No. AF319542) plasmid was that obtained from mouse kidney^36^. The cDNAs was subcloned into the pCR3.1 vector. Mutations were made by site-directed mutagenesis into the pCR3.1 vector as previously described^69^ and confirmed by sequencing. HEK-293 cells were cultured in Dulbecco’s modified Eagle’s medium (DMEM) and F12 Ham’s medium at a 1:1 ratio and supplemented with 10% fetal calf serum at 37 °C in 5% CO_2_. For electrophysiological experiments, transient transfections were performed by electroporation, using CD8 cotransfection to identify effectively transfected cells. The CD8 antigen was revealed with microspheres (Dynabeads) coated with an anti-CD8 antigen. Experiments were performed 4–8 h after electroporation. In experiments with the voltage-sensitive phosphatase TASK-2 or its mutants were co-electroporated with a pCR3.1-DrVSP-IRES-CD8 vector.

### Electrophysiological assays

Transiently transfected HEK-293 cells in 35 mm Petri dishes were transferred to the stage of an inverted microscope for study. There they were continuously superfused with a bathing solution containing (in mM): 67.5 Na_2_SO_4_, 4 KCl, 1 K gluconate, 2 CaCl_2_, 1 MgCl_2_, 105 sucrose, and 10 Hepes/Tris (pH 7.5) (standard solution). A high K^+^ solution was obtained by equimolar replacement of Na^+^ by K^+^. The pipette solution contained (mM) 8 KCl, 132 potassium gluconate, 1 MgCl_2_, 10 EGTA, 1 Na_3_ATP, 0.1 GTP, 10 HEPES/Tris, pH 7.4. Standard whole-cell, patch-clamp recordings were performed as described elsewhere^70^. All our whole-cell experiments keep both intra- and extracellular chloride at 10 mM and although currents were measured at several potentials, data mostly reported are those obtained at 0 mV. Potentials were corrected for liquid junction shifts. Acquisition and analysis was done with Clampfit 9.0 (Axon Instruments, Foster City, CA, USA). Further data analysis was done using the curve fitting features of SigmaPlot v. 12 (Systat Software Inc., San Jose, CA, USA).

Recordings in the inside-out isolated patch configuration were performed in the gap-free mode at −60 mV. The inside aspect of the patch was bathed with a solution containing in mM: 10 KCl, 10 Glucose, 1 MgCl_2_, 10 EGTA, 1 Na_3_ATP, 0.1 GTP, 6.8 g/100 ml Sucrose, 10 Hepes/Tris, pH 7.4. The pipette contained in mM: 140 KCl, 10 Glucose, 6.8 g/100 ml Sucrose, 10 Hepes/Tris pH 7.4. For isolated patches, borosilicate capillaries (Harvard Apparatus GC150F-10 1.5 ODx0.86 ID) were fire polished to 4–5 MΩ and covered in beeswax. Acquisition was at 10 kHz with filter at 3 kHz (4-pole Bessel) and analysis was done with Clampfit 9.0 (Axon Instruments, Foster City, CA, USA). Traces shown are prints from crude Clampfit screen shots and quantification was done using the average currents were calculated at intervals using Clampfit after baseline adjustment.

### Materials

MTSET, [2-(trimethylammonium)ethyl] methanethiosulfonate chloride was obtained from Santa Cruz Biotechnology; phosphatidylinositol 4-monophosphate diC8, phosphatidylinositol 4,5-bisphosphate diC8, phosphatidylinositol 3,4-bisphosphate diC8, and phosphatidylinositol 3,4,5-trisphosphate diC8, as well as 1,2 dioctanoyl-*sn*-glycerol, were purchased from Avanti Polar Lipids. All other chemicals were purchase from Sigma Chemical Company.

## Additional Information

**How to cite this article**: Niemeyer, M. I. *et al*. Phosphatidylinositol (4,5)-bisphosphate dynamically regulates the K_2P_ background K^+^ channel TASK-2. *Sci. Rep.* **7**, 45407; doi: 10.1038/srep45407 (2017).

**Publisher's note:** Springer Nature remains neutral with regard to jurisdictional claims in published maps and institutional affiliations.

## Supplementary Material

Supplementary Information

## Figures and Tables

**Table 1:** Functional effect of cysteine mutations in certain TASK-2 positively-charged residues.

Mutation	I at 80 mV	MTSET effect	VSP inhibition	Neomycin inhibition
R4C	✓	—	—	undet
K254C	✓	✓	—	undet
K297C	✓	✓	✓	undet
R438C	—	—	✓	✓

✓ under “I at 80 mV” indicates a significantly decreased whole cell-current with respect to WT TASK-2. ✓ under “MTSET effect” indicates a significant increase in current after treatment with the methanesulfonate reagent. ✓ under “VSP inhibition” reveals an accelerated inhibition of the mutant compared with the speed of inhibition of WT TASK-2 by activation of cotransfected DrVSP. ✓ under Neomycin inhibition indicates a significantly lower IC_50_ for inhibition by neomycin compared to control. — in all cases stands for no significant effect. Undet, undetermined.
