# Supplementary material for: Phosphatidylinositol (4,5)-bisphosphate dynamically regulates the K2P background K+ channel TASK-2
Source: Sci Rep. 2017 Mar 30;7:45407. doi: 10.1038/srep45407 (PMC5371824; doi:10.1038/srep45407)
Supplement: Supplementary Information [file srep45407-s1.pdf]

Supplementary Information.

Phosphatidylinositol (4, 5)-bisphosphate dynamically regulates the K_{2P} background K^+ channel TASK-2.

María Isabel Niemeyer[1], L. Pablo Cid[1], Marc Paulais[2], Jacques Teulon[2] & Francisco V. Sepúlveda[1]

[1]Centro de Estudios Científicos (CECs), Avenida Arturo Prat 514, Valdivia, Chile; [2]UPMC Université Paris 06, UMR_S 1138, Team 3 and INSERM, UMR_S 872, Paris, France

Correspondence:

Francisco V. Sepúlveda
Centro de Estudios Científicos (CECs)
Avenida Arturo Prat 514
5110466 Valdivia
Chile
fsepulveda@cecs.cl

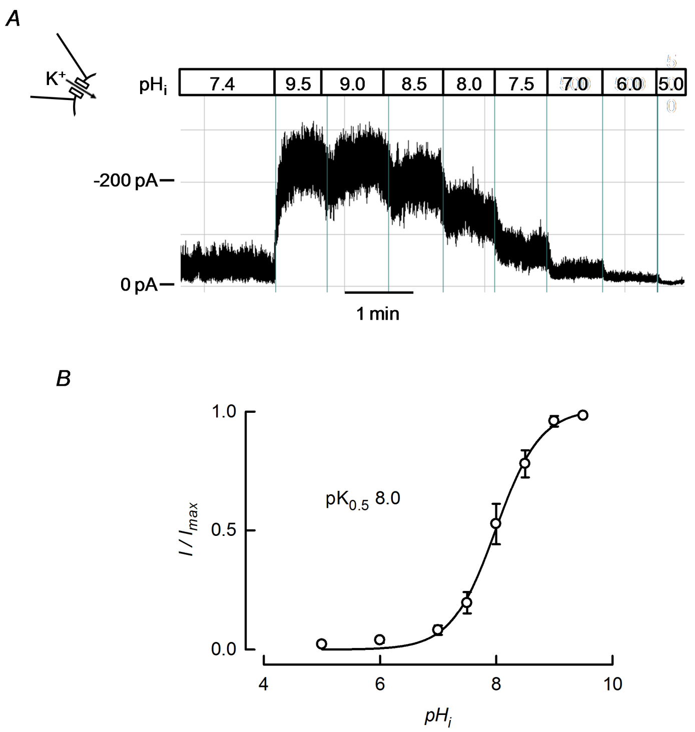

Fig. S1. Intracellular pH dependence on the current in excised inside-out patches of membrane obtained from HEK-293 cells transfected with TASK-2 cDNA. Recordings shown in A were done exactly as described in the Methods section of the main text. Changes in pH were implemented using the buffers described previously[1]. In B the dependence of the current upon intracellular pH is shown. The solid line is a fit of a Hill equation to the data that yielded a $pK_{0.5}$, the pH at which half of the maximal activity is obtained, of 8.0 ± 0.04 and n_H of 1.14 ±0.10. The data are means ± SEMs of four separate experiments. Those values do not differ from previously published figures for $pK_{0.5}$ and n_H of 8.0 and 0.9 respectively, for TASK-2 pH_i-dependence measured in whole-cell recordings of TASK-2-expressing cells[2].

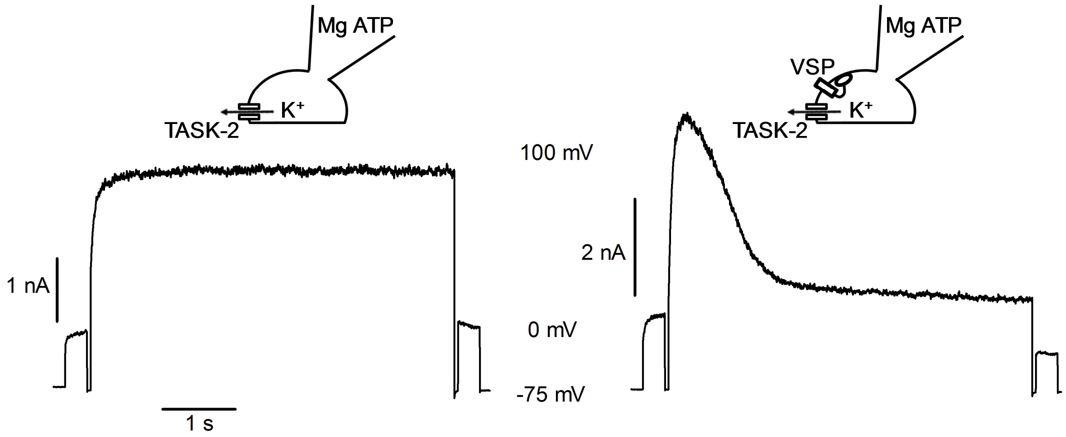

Fig. S2. Effect of prolonged depolarisation on TASK-2-mediated current in the presence or absence of cotransfected DrVSP. Solutions and other details are as in Fig. 1A of the main text.

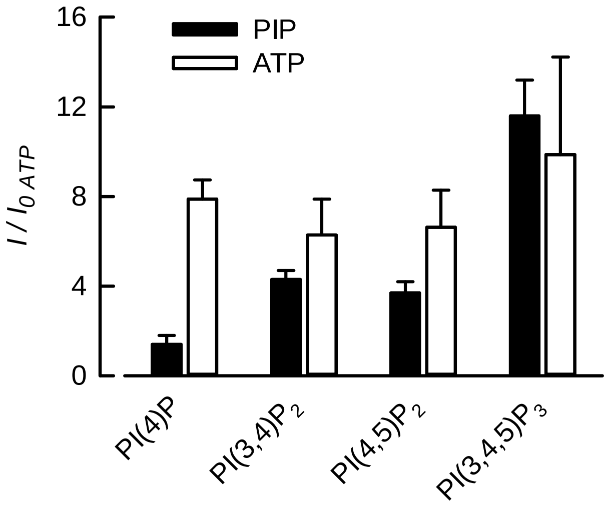

Fig. S3. Comparison of the maximal effect on TASK-2-mediated current of various phosphoinositides measured in the absence of ATP, with that of 1 mM ATP added thereafter. The filled columns report the maximal effects of the PIPs obtained from the fits in Fig. 5E of the main text. Empty columns show the effect of ATP addition. Data are derived from 3-6 replicates.

Using MTSET, [2-(Trimethylammonium)ethyl] Methanethiosulfonate, to modify intracellularly-facing cysteine residues in TASK-2.

We introduced cysteines to replace potentially intracellularly-facing basic residues by side-directed mutagenesis. Modification with MTSET in excised, inside-out patches was used in an attempt to restore the positive charge at the site. This experiment requires, however, that no endogenous cysteine is modified in the WT protein with a measurable functional consequence for the channel. This was not the case with TASK-2, whose activity was markedly reduced by MTSET (Fig. S4). That the inhibition was probably mediated by modifying a native cysteine is suggested by the reversibility of the effect by treatment with dithothreitol (DTT).

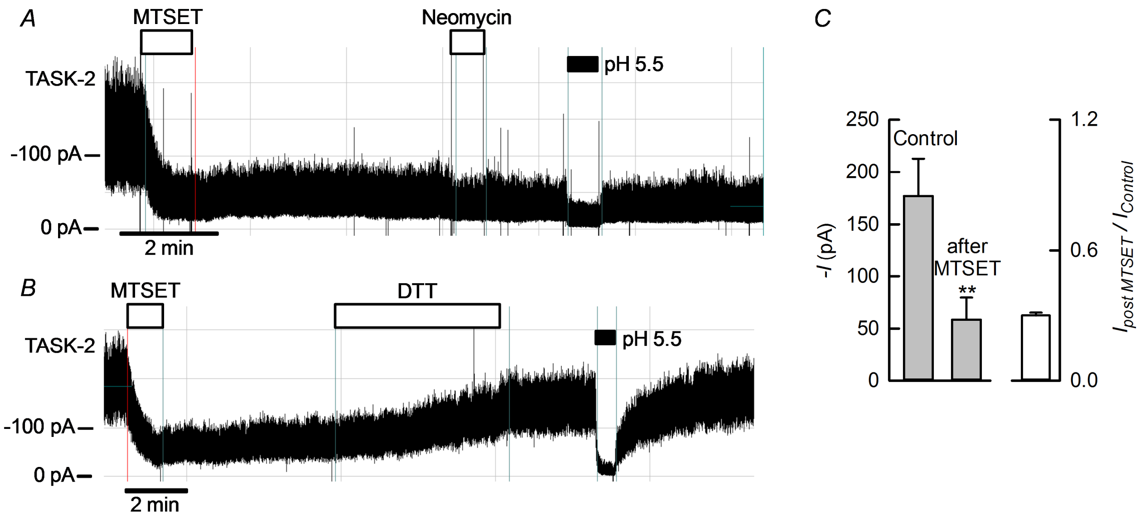

Fig. S4. Recording from excised inside-out patches of membrane taken from a HEK-293 cells over-expressing TASK-2. A and B: two traces from different patches of membrane are shown. The patches were held at -60 mV in the presence of 1 mM ATP. At the intervals indicated by the boxes the intracellular aspect of the membrane was exposed to 1 mM MTSET, and to 300 µM Neomycin or pH 5.5. In the patch shown in the lower trace, the reducing agent DTT was also added at 2 mM. C: Summary of experiments comparing the current before and after treatment with MTSET. **: P<0.01 by paired t-test.

We next searched for the cysteine(s) responsible for the MTSET-modification inhibition of TASK-2 by mutating those putatively facing intracellularly (Fig. S5).

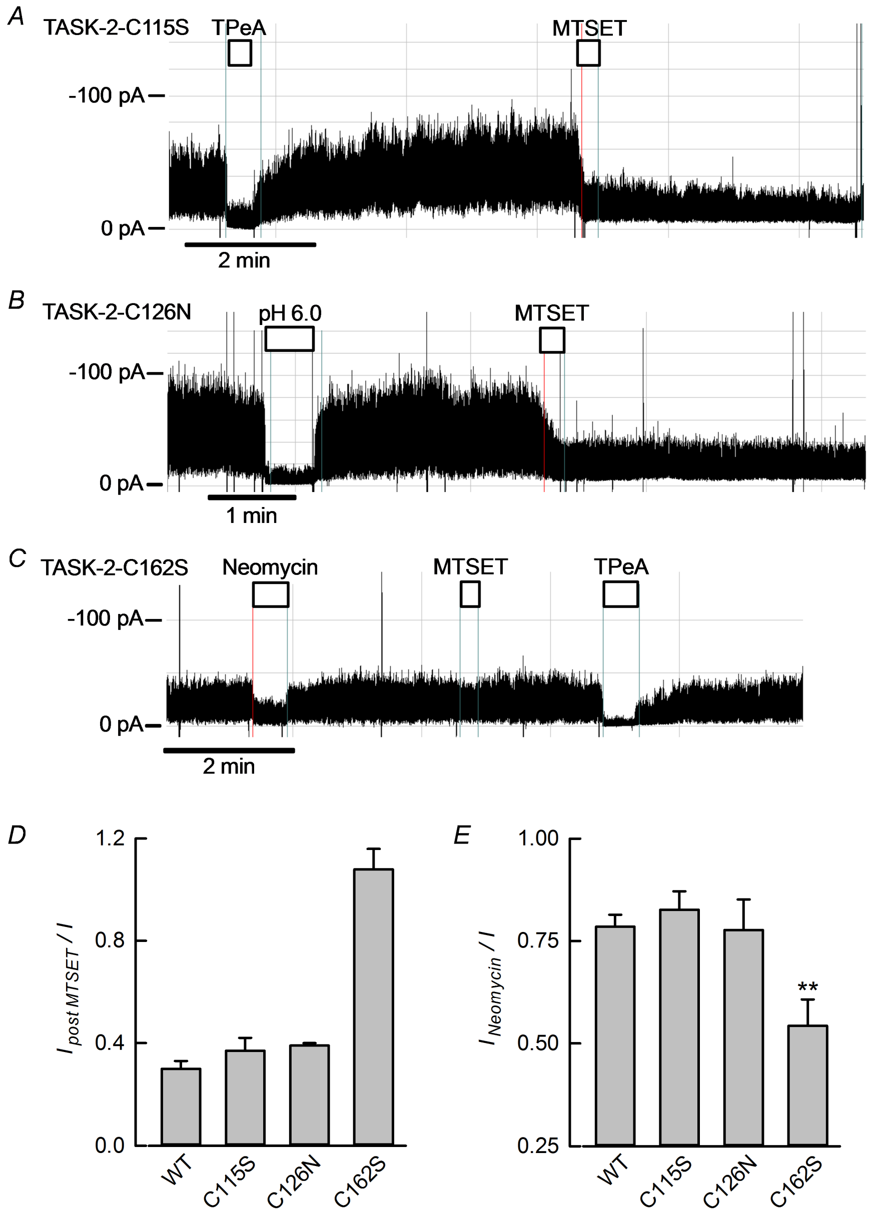

Fig. S5. Effect of MTSET on mutant TASK-2 with invalidated cysteines potentially facing the intracellular side of the membrane. A-C: Current traces for the different mutants (details as in Fig. S4). Of the three potentially cysteines attacked by MTSET, C115, C126 and C162, only TASK-2-C162S showed lack of MTSET effect. (D). This mutant expressed only small currents and also had significantly diminished neomycin sensitivity when compared with that of WT TASK-2 (E, **: P<0.01 by ANOVA). Columns are means ± SEMs (n= 4-11).

As seen in Fig. S5, MTSET sensitivity was conferred to TASK-2 exclusively by the presence of residue C162. Its invalidation as a reducible side-chain by replacement by serine led to small currents and a decrease in sensitivity to neomycin, perhaps indicating a change in the interaction TASK-2-IP(4,5)P_2.

In searching for a way to protect WT TASK-2 of the attack of MTSET we stumbled upon the property of the intracellular pore blocker TPeA to efficiently prevent against any effect of this cysteine reagent on the channel (Fig. S6). As TPeA acts deeply within the inner pore of K_{2P} channels we assume it will have no effect on residues that potentially will interact with the head group of IP(4,5)P2 presumably in the inner leaflet of the membrane and therefore remote from the inner pore. Although we ignore the mechanism mediating the TPeA protection of TASK-2 activity from MTSET modification, the manoeuvre illustrated in Fig. S6 allows the use of the reducing agent without any measurable lasting effect on the WT channel.

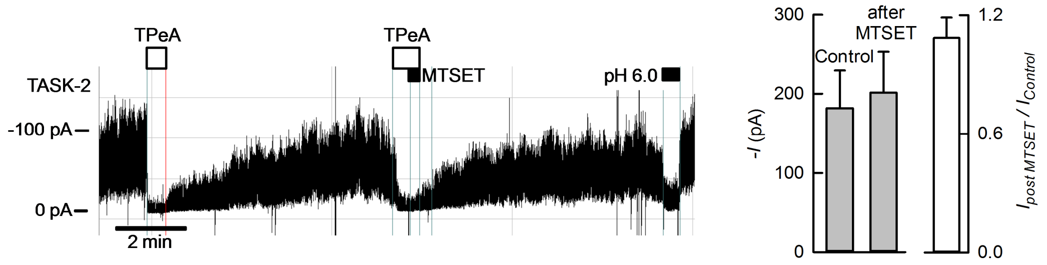

Fig. S6. TPeA protects WT TASK-2 from inhibition by MTSET. Details as in Fig. S2. Columns are means ± SEMs (n= 11).

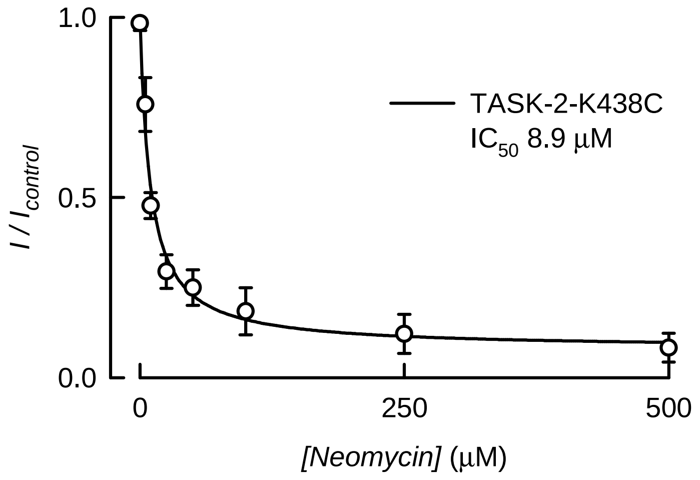

Fig. S7. Concentration ependence for the inhibition of TASK-2 mutant R438C by intracellular neomycin. Recording were obtained from excised inside-out patches of membrane taken from a HEK-293 cells over-expressing TASK-2-R438C. The patches were held at -60 mV in the presence of 1 mM intracellular ATP. Neomycin was increased stepwise from 5 to 500 µM in the solution bathing the intracellular aspect of the membrane. The graph the effect of neomycin and are the means of four experiments that gave an average IC_{50} for neomycin of 8.9 ± 1.5 µM (mean ± SEM, n=4).

References

1 Niemeyer, M. I. *et al.* Neutralization of a single arginine residue gates open a two-pore domain, alkali-activated K^+ channel. *Proc. Natl. Acad. Sci. U. S. A.* **104**, 666-671 (2007).

2 Niemeyer, M. I., Cid, L. P., Peña-Münzenmayer, G. & Sepúlveda, F. V. Separate gating mechanisms mediate the regulation of K_{2P} potassium channel TASK-2 by intra- and extracellular pH. *J. Biol. Chem.* **285**, 16467-16475 (2010).
